# CoV-Seq, a New Tool for SARS-CoV-2 Genome Analysis and Visualization: Development and Usability Study

**DOI:** 10.2196/22299

**Published:** 2020-10-02

**Authors:** Boxiang Liu, Kaibo Liu, He Zhang, Liang Zhang, Yuchen Bian, Liang Huang

**Affiliations:** 1 Baidu Research Sunnyvale, CA United States; 2 School of Electrical Engineering & Computer Science Oregon State University Corvallis, OR United States

**Keywords:** COVID-19, SARS-CoV-2, bioinformatics, genetics, genome, virus, sequence, data sets, programming, web server

## Abstract

**Background:**

COVID-19 became a global pandemic not long after its identification in late 2019. The genomes of SARS-CoV-2 are being rapidly sequenced and shared on public repositories. To keep up with these updates, scientists need to frequently refresh and reclean data sets, which is an ad hoc and labor-intensive process. Further, scientists with limited bioinformatics or programming knowledge may find it difficult to analyze SARS-CoV-2 genomes.

**Objective:**

To address these challenges, we developed CoV-Seq, an integrated web server that enables simple and rapid analysis of SARS-CoV-2 genomes.

**Methods:**

CoV-Seq is implemented in Python and JavaScript. The web server and source code URLs are provided in this article.

**Results:**

Given a new sequence, CoV-Seq automatically predicts gene boundaries and identifies genetic variants, which are displayed in an interactive genome visualizer and are downloadable for further analysis. A command-line interface is available for high-throughput processing. In addition, we aggregated all publicly available SARS-CoV-2 sequences from the Global Initiative on Sharing Avian Influenza Data (GISAID), National Center for Biotechnology Information (NCBI), European Nucleotide Archive (ENA), and China National GeneBank (CNGB), and extracted genetic variants from these sequences for download and downstream analysis. The CoV-Seq database is updated weekly.

**Conclusions:**

We have developed CoV-Seq, an integrated web service for fast and easy analysis of custom SARS-CoV-2 sequences. The web server provides an interactive module for the analysis of custom sequences and a weekly updated database of genetic variants of all publicly accessible SARS-CoV-2 sequences. We believe CoV-Seq will help improve our understanding of the genetic underpinnings of COVID-19.

## Introduction

Since its identification in late 2019, the novel coronavirus SARS-CoV-2 has caused an outbreak of viral pneumonia and has become a global pandemic. Despite efforts to contain its spread, as of late September 2020, SARS-CoV-2 had infected nearly 33 million patients and caused nearly 1 million deaths worldwide [1]. To understand its evolution and genetics, scientists have sequenced SARS-CoV-2 genomes from patients across different age groups, genders, ethnicities, locations, and disease stages [2]. These genomic sequences are being shared on public repositories at a rapid pace, with thousands of new sequences every week [3-5]. To keep up with the latest developments, scientists need to frequently download and clean new data sets, which is an ad hoc and time-consuming process. Furthermore, scientists with limited knowledge of bioinformatics or programming may experience difficulty in analyzing SARS-CoV-2 genomes.

We developed the CoV-Seq toolkit to address these challenges. CoV-Seq consists of several components: a data analysis pipeline that takes FASTA sequences and generates variant callsets in variant call format (VCF) and open reading frame (ORF) predictions. The pipeline automatically filters low-quality sequences, removes duplicate sequences, performs sequence alignment, and identifies and annotates genetic variants. We provide a web server [6] to enable the rapid analysis of custom sequences without any programming. The web interface includes an interactive genome visualizer and tabulated displays of genetic variants and ORF predictions. All results can be downloaded for downstream analysis. Further, we provide a command-line interface to allow high-throughput processing in local environments. To facilitate data sharing, we aggregate SARS-CoV-2 sequences from the Global Initiative on Sharing Avian Influenza Data (GISAID) [3], National Center for Biotechnology Information (NCBI) [4], European Nucleotide Archive (ENA) [5], and China National GeneBank (CNGB).

## Methods

### Data Collection

The majority of publicly available SARS-CoV-2 genomic sequences are deposited into the following databases: GISAID (Multimedia Appendix 2 lists GISAID contributors), NCBI, ENA, and CNGB. All databases provide the option of downloading data in a batch. We used Selenium [7] to automate the data download process.

### Data Preprocessing

**Figure 1:**
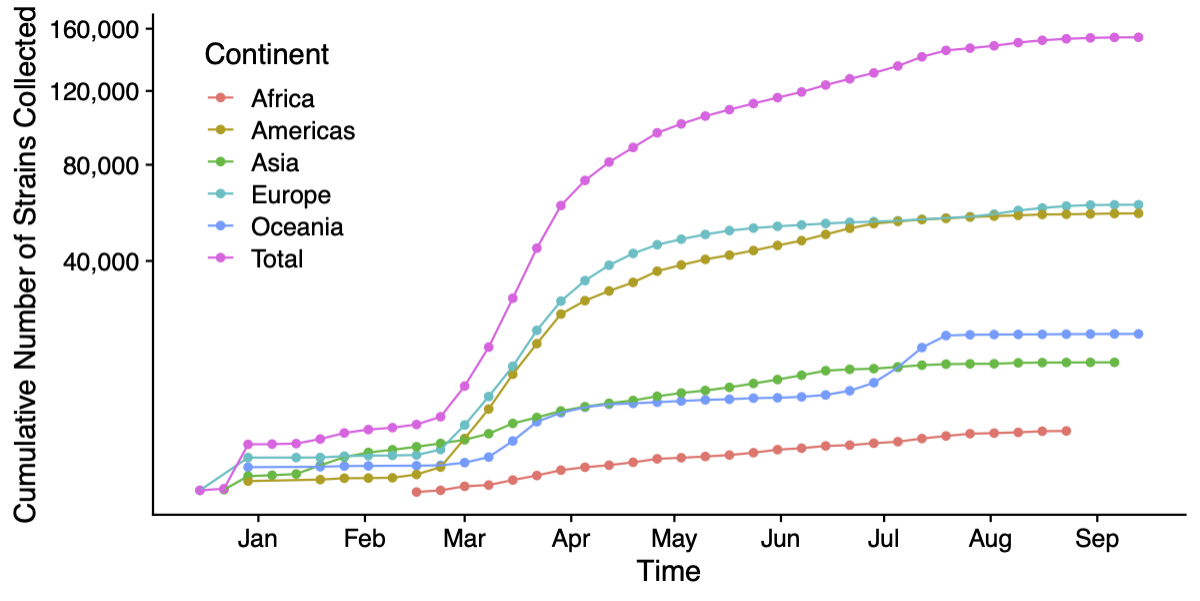
The cumulative number of sequences hosted by public databases has increased over time. Sequences from Asia increased steadily since January, whereas sequences from other continents saw a dramatic increase in March. Each data point represents a week. Note that the x-axis shows collection dates, which can precede submission dates by several weeks (eg, a sequence collected in July may be submitted in August). Therefore, lines that do not reach August indicate that sequences recently collected have not been submitted yet.

We aggregated SARS-CoV-2 sequences from GISAID, NCBI, ENA, and CNGB. Many sequences represented incomplete genomes, sometimes containing only a single gene. We filtered these genomes using a lenient cutoff of 25,000 nucleotides because doing so removed distinctly incomplete genomes while retaining complete genomes (Figure 1 in Multimedia Appendix 1). Both NCBI and ENA are part of the International Nucleotide Sequence Database Collaboration (INSDC) and therefore contain duplicate submissions, which we removed by comparing the accession IDs. Further, dual submissions can appear in both GISAID and INSDC under different accession IDs. We considered two submissions as suspect duplications if they had identical genomic sequences. These suspect duplications were marked in the metadata but not removed because a strain can infect multiple patients.

### Pairwise Sequence Alignment

We performed pairwise alignment against the reference sequence (NCBI accession ID: NC_045512.2) using Multiple Alignment using Fast Fourier Transform (MAFFT) [8] with default options.

### Variant Calling

**Figure 2:**
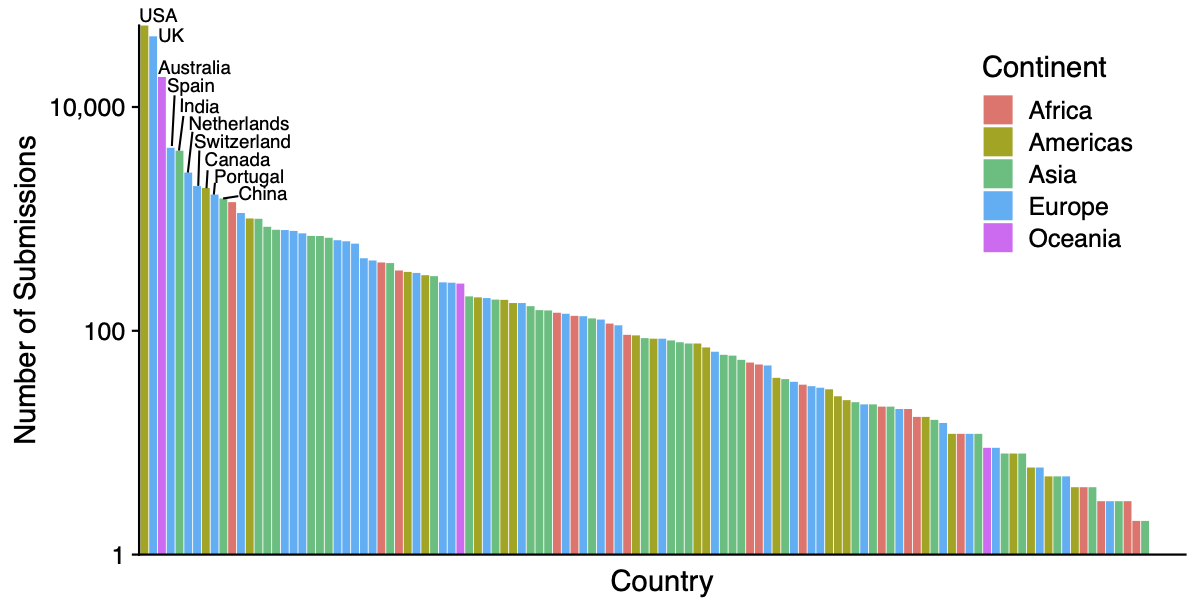
The number of submissions by country. The ten countries with the highest numbers of submissions are marked.

**Figure 3:**
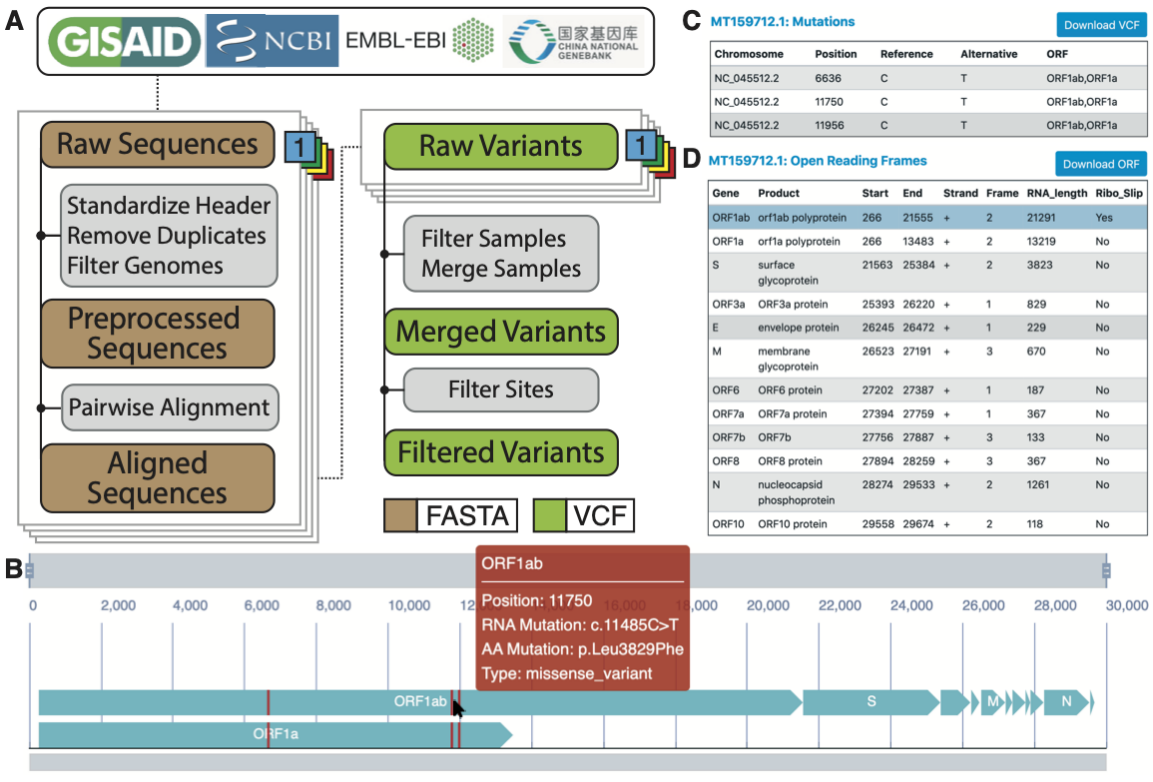
The CoV-Seq pipeline and web interface. (A) Genomic sequences are collected from GISAID, NCBI, ENA, and CNGB. We remove incomplete genomes (length <25,000 nucleotides) and duplicate genomes before alignment with MAFFT against the reference genome NC_045512.2. We use a custom Python script to generate raw variant calls and remove samples with too many mutations, indicative of sequencing error. After merging VCFs, we remove multiallelic sites and variants with the poly-A tail for a filtered set of variants. (B) The interactive genome visualizer shows ORFs (turquoise) and mutations (red). Users can zoom with the top bar and pan with the bottom bar. Hovering over ORF bodies and mutations will trigger pop-up windows for relevant information. (C) The mutation table shows positions, alleles, and intersecting ORFs. (D) The ORF table shows predicted ORF boundaries and supporting information. CNGB: China National GeneBank; ENA: European Nucleotide Archive; GISAID: Global Initiative on Sharing Avian Influenza Data; MAFFT: Multiple Alignment using Fast Fourier Transform; NCBI: National Center for Biotechnology Information; ORF: open reading frame; VCF: variant call format.

We used a custom Python script for variant calling, in which we considered single nucleotide polymorphisms (SNPs), insertions, and deletions. We left-normalized each variant with bcftools [9] and removed samples with too many variants, indicative of sequencing error. We used a lenient cutoff of 350 variants because doing so removed samples with extremely large numbers of variants while keeping most samples (Figure 2 in Multimedia Appendix 1). During postprocessing, we removed multiallelic sites because these sites were more likely to occur in regions prone to sequencing error, such as the two ends of the genome (Figure 3 in Multimedia Appendix 1). Further, we removed variants within the poly-A tail. The filtered variant callset was annotated with snpEff [10].

### ORF Boundary Detection

To detect ORF boundaries, we employed a method similar to Viral Annotation Pipeline and iDentification (VAPiD) [11]. Since NCBI Genbank provides genetic annotations for the SARS-CoV-2 reference genomes, we translated the coordinates of ORF boundaries from the reference genome to the query genome using their pairwise alignment. For multisegment ORFs (ie, due to ribosomal slippage), we annotated each segment independently and combined them afterward.

### Interactive Visualization

The CoV-Seq web server is hosted on Amazon Web Services (AWS) Elastic Beanstalk with load balancer enabled, running with the Flask framework [12], Jinja template engine [13], and Werkzeug [14] Web Server Gateway Interface (WSGI) toolkit. After the user submits data through either a text box (for a single sequence) or file upload (for an arbitrary number of sequences), the back-end program will perform pairwise sequence alignment, variant calling, and ORF boundary detection to generate results in VCF and JSON formats. The front-end templates will then render genome sequences on a new page using JSON data, the ECharts [15] library, and dedicated designed JavaScript functions. The result page highlights mutations on submitted sequences against the reference sequence and shows details upon cursor hover. Users can also zoom in to check the details of the genomic sequence. Selecting a specific ORF will expand the display to show the mutation table, the ORF table, and the gene sequence for the selected ORF.

## Results

### Statistics on Collected Sequences

The number of SARS-CoV-2 sequences submitted to GISAID, NCBI, ENA, and CNGB has increased over time. Figure 1 shows the cumulative number of sequences by collection date. Sequences from Asia increased steadily since January, whereas sequences from other continents initially grew slowly but saw a dramatic increase in pace in March. The submission volume reflects the global spread of the virus, starting with the initial outbreak within China in January and followed by the subsequent global outbreak in March. Note that although the number of submissions correlates with the number of cases overall, the number of submissions does not reflect the actual case numbers (Figure 2). The ten countries with the highest number of submissions include five European countries (United Kingdom, Spain, Portugal, the Netherlands, and Switzerland), two Asian countries (India and China), two North American countries (United States and Canada), and one Oceanian country (Australia).

### Interactive Visualization With Custom Sequences

CoV-Seq provides an intuitive web interface (Figure 3A) for analyzing and visualizing SARS-CoV-2 variants and ORFs. Upon receiving custom sequences, CoV-Seq identifies ORF boundaries and genetic variants and displays them alongside a full-length genome (Figure 3B). Users interact with the genome by dragging the zoom bar to adjust the magnification and the position bar to pan along the genome. When the genome window contains less than 150 nucleotides, letters will appear to indicate both the nucleotide bases and amino acid residues. Hovering over ORF bodies or variants will trigger pop-up windows for relevant information (Figure 3B, red box). Clicking on an ORF brings up three tables. The first table shows all variants and their positions, alleles, and ORFs in which they belong (Figure 3C). A second table shows ORF annotations obtained by aligning input sequences against the reference sequence and transferring annotations from Genbank (Figure 3D). A third table shows nucleotide and protein sequences for the selected ORF (not shown). All tables can be downloaded for further analysis.

### CoV-Seq Command-Line Interface

Due to the rapid accumulation of SARS-CoV-2 genomic sequences, a point-and-click web interface is time-consuming for large collections. Therefore, we provide a command-line interface (CLI) for high-throughput processing of sequence batches [16]. The CLI allows the user to identify variants and ORF boundaries from multiple sequences with a single command. Further, the CLI allows the user to identify variants from multiple sequences from a FASTA file with one command.

### Downloading Analysis-Ready Data

To facilitate downstream analysis with publicly available data, we aggregated SARS-CoV-2 genomic sequences from GISAID, NCBI, ENA, and CNGB; we also identified and annotated genetic variants. In addition, we aggregated metadata with key information such as the location and collection date for each sequence (see Methods). All aggregated information can be downloaded from the CoV-Seq web server [17]. Based on this set of information, we provide statistics on the geographical and chronological distributions of sequence submissions and encourage further analysis by other scientists. 

## Discussion

### Principal Results

In this paper, we have described CoV-Seq, a web server that enables the rapid analysis of SARS-CoV-2 genomic sequences. CoV-Seq consists of several components. The interactive visualization module accepts custom sequences as input and displays the genetic variants and ORF boundaries on an interactive genome browser. For batch processing needs, CoV-Seq provides a CLI interface that processes many sequences at once. To encourage downstream analysis with publicly available data, CoV-Seq provides downloadable analysis results (updated weekly) with sequence metadata and genetic mutations.

### Limitations

CoV-Seq is currently limited to SARS-CoV-2 sequences. The web server does not allow custom reference sequences other than SARS-CoV-2. We chose to focus on this virus because it has constituted the majority of processing requests during the COVID-19 pandemic. We plan to provide additional functionality to accept custom reference sequences in a future release.

### Comparison With Prior Work

The existing software packages VAPiD [11] and Viral Genome ORF Reader (VIGOR) [18] focus on gene annotations. To our knowledge, a software package that identifies, annotates, and visualizes genetic variants of SARS-CoV-2 has not previously been created.

### Conclusions

We developed the CoV-Seq web server for fast and easy analysis of SARS-CoV-2 sequences. We hope CoV-Seq will help improve our understanding of the genetics of COVID-19. In the future, we plan to expand the scope of CoV-Seq to include other viruses.

## Appendix

Multimedia Appendix 1 Supplementary figures.

Multimedia Appendix 2 Acknowledgment table for GISAID contributors.

## Abbreviations

CLI: command-line interface
GISAID: Global Initiative on Sharing Avian Influenza Data
NCBI: National Center for Biotechnology Information
ENA: European Nucleotide Archive
CNGB: China National GeneBank
ORF: open reading frame
VCF: variant call format
