# Supplementary material for: CoV-Seq, a New Tool for SARS-CoV-2 Genome Analysis and Visualization: Development and Usability Study
Source: J Med Internet Res. 2020 Oct 2;22(10):e22299. doi: 10.2196/22299 (PMC7537720; doi:10.2196/22299)
Supplement: Multimedia Appendix 1 [file jmir_v22i10e22299_app1.pdf]

Supplemental Material for CoV-Seq: SARS-CoV-2 Genome Analysis and Visualization

September 13, 2020

1 Supplementary Figures

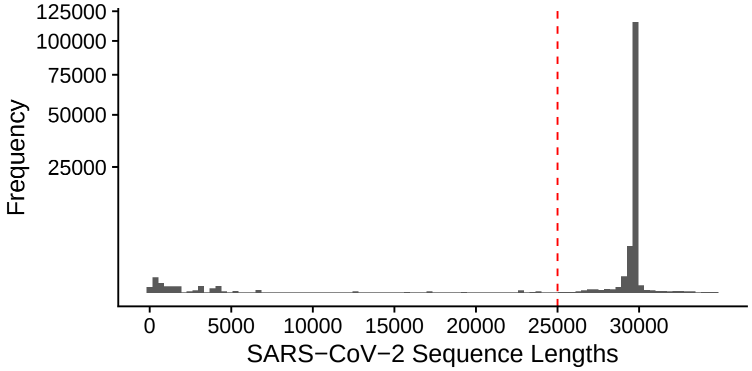

Figure S1: Distribution of SARS-CoV-2 sequence lengths. Sequences with lengths less than 25,000 nucleotides are removed during preprocessing.

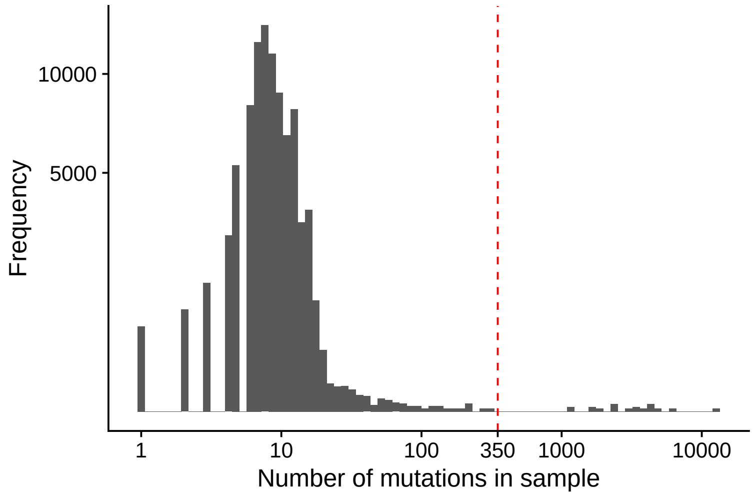

Figure S2: Distribution of sample mutations identified against the reference genome NC_045512.2. Samples with more than 350 mutations are removed during post-processing.

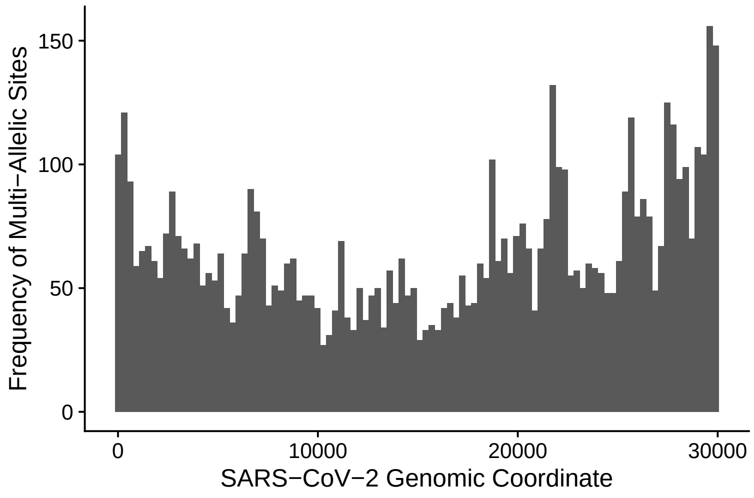

Figure S3: Distribution of multi-allelic sites along the SARS-CoV-2 genome. Multi-allelic sites are more likely to be identified at the beginning and the end of the genome.
